# Metallo-polyelectrolytes as a class of ionic macromolecules for functional materials

**DOI:** 10.1038/s41467-018-06475-9

**Published:** 2018-10-18

**Authors:** Tianyu Zhu, Ye Sha, Jing Yan, Parasmani Pageni, Md Anisur Rahman, Yi Yan, Chuanbing Tang

**Affiliations:** 1 Department of Chemistry and Biochemistry, University of South Carolina, Columbia, South Carolina 29208 USA; 2 Department of Applied Chemistry, School of Science, Northwestern Polytechnical University, Xi’an, Shannxi 710129 China

## Abstract

The fields of soft polymers and macromolecular sciences have enjoyed a unique combination of metals and organic frameworks in the name of metallopolymers or organometallic polymers. When metallopolymers carry charged groups, they form a class of metal-containing polyelectrolytes or metallo-polyelectrolytes. This review identifies the unique properties and functions of metallo-polyelectrolytes compared with conventional organo-polyelectrolytes, in the hope of shedding light on the formation of functional materials with intriguing applications and potential benefits. It concludes with a critical perspective on the challenges and hurdles for metallo-polyelectrolytes, especially experimental quantitative analysis and theoretical modeling of ionic binding.

## Abstract (web summary)

Polyelectrolytes are a class of macromolecules containing charged groups. Here the authors review properties and functions of metallo-polyelectrolytes to shed light on how to harness fundamental physiochemical properties for applications in biomedical sciences and advanced materials.

## Introduction

Polyelectrolytes are a class of macromolecules containing charged groups, either positively, negatively or both^1^. Equally important to uncharged neutral polymers, polyelectrolytes have been widely used for a myriad of applications, ranging from genetic coding to synthetic membranes for separation to drug delivery, to name just a few^2–4^. Quaternary ammonium-based cationic and organic acid-based anionic polymers are among the most common polyelectrolytes. While polyelectrolytes continue to attract attention in the areas of chemistry, physics, engineering, biology, and pharmaceutics, it is appealing for both academia and industry to design charged macromolecules toward unprecedented properties and functions to address problems that the world is facing regarding health, energy, environments and sustainability in the 21st century^5–8^.

The fields of polymer and macromolecular sciences have enjoyed a unique combination of metals and soft organic frameworks in the name of metallopolymers, metal-containing polymers or organometallic polymers since the middle of 20th century^9–12^. When metallopolymers carry charged groups, they form a class of polyelectrolytes or metallo-polyelectrolytes. This is an emerging area that is particularly well suited for manufacturing functional materials. For convenience in this review, we refer traditional all-organic polyelectrolytes as to organo-polyelectrolytes.

**Fig. 1:**
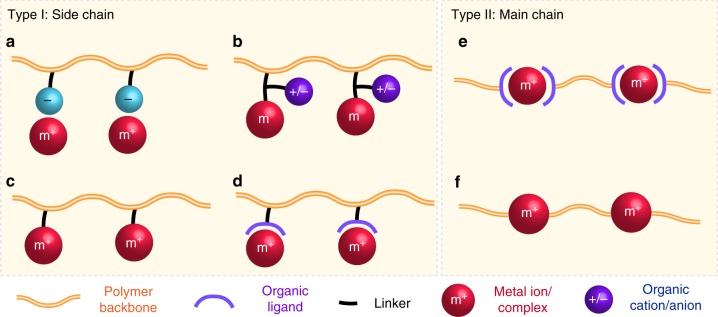
Diverse topologies of metallo-polyelectrolytes. **a** metal ions attached to the side chain of organic polyelectrolytes through electrostatic interaction; **b** neutral metal complexes at the side chain of polyelectrolytes; **c** charged metal complexes at the side chain by covalent bonding; **d** charged metal complexes at the side chain through coordination; **e** main chain metallo-polyelectrolytes formed by coordination; **f** main chain metallo-polyelectrolytes formed by covalent bonding

Depending on the metal location and the nature of chemical bonding of metals with organic frameworks, metallo-polyelectrolytes have a variety of topologies as summarized in Fig. 1. In the case of metals as a pendant group, metal cations can be counterions to anionic polyelectrolytes (Fig. 1a). Polyelectrolyte frameworks could also carry neutral metal centers (Fig. 1b). In both cases, the charged groups in these polymers are still all organic, thus not being further discussed in this review unless mentioned specifically. On the other hand, metal cations can coordinate or covalently complex at the side chain to form metallo-polyelectrolytes (Fig. 1c, d). Similarly, main-chain metallo-polyelectrolytes can be also fabricated via coordination or covalent bonding (Fig. 1e, f). Organo-polyelectrolytes generally utilize covalent bonding to form ionic charged groups as an integral part of macromolecules. However, the formation of ionic centers via coordination chemistry is rare in this kind of polyelectrolytes.

**Fig. 2:**
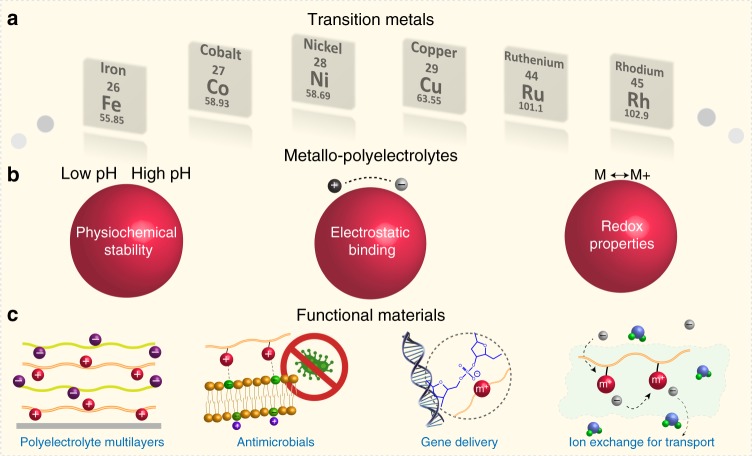
An overarching summary to illustrate a few key functions of metallo-polyelectrolytes. **a** most common transition metals for building metallo-polyelectrolytes; **b** a few properties of metallo-polyelectrolytes including electrostatic binding, redox and physiochemical stability; **c** applications in the areas of polyelectrolyte multilayers, antimicrobials, gene delivery and ion exchange for transport

Based on different architectures of metallo-polyelectrolytes, there are two major approaches to integrate metals into macromolecules: (1) metal ions attached to conventional polyelectrolytes through electrostatic interaction (Fig. 1a) or neutral metals (or metal complexes) added onto conventional polyelectrolytes (Fig. 1b), in which the charged groups behave most likely the same way as organo-polyelectrolytes, though the metals could provide additional functions; (2) charged metals integrated into organic frameworks (Fig. 1c–f), in which the properties of polyelectrolytes would be mostly dictated by the ionic metal centers. The latter scenario is expected to be substantially different from traditional organo-polyelectrolytes. As shown in Fig. 2, this short review articulates properties and functions of the second type of metallo-polyelectrolytes, in the hope of shedding light on how to harness their fundamental physiochemical properties for applications in biomedical sciences and advanced materials, as well as to present the most exciting discoveries for the broad communities of polyelectrolytes and macromolecules. Particularly, some of our recent research efforts are elicited to exemplify the benefits. While there are a few recent reviews on metal-containing polymers^8,11,13–15^, an examination on emerging directions of metallo-polyelectrolytes has not been done, to the best of our knowledge. We organize this review according to the following: (1) electronic, bonding, and redox properties; (2) functional materials via redox or electrostatic interactions; (3) ion-exchange for transport.

## Electronic, bonding, and redox properties

Polyelectrolytes are ionic macromolecules that can complex with a variety of oppositely charged substrates. The ionic groups and polymeric backbone are the most important components for polyelectrolytes. When mixing with substrates carrying oppositely charged ions, it typically encounters enthalpy-driven electrostatic interactions. The association between oppositely charged molecules simultaneously initiates entropic changes by the loss of counterions. Most often, the entropic change drives the complexation, while the attractive Coulombic interactions between opposite charges are favorable in terms of enthalpy change. On the other hand, complexes between two oppositely charged polyelectrolytes, often referred to as polyelectrolyte complexes (PECs), are mostly studied^16,17^. Depending on the strength of complexation, PECs often undergo phase separation, with the formation of either solid precipitates or liquid-like coacervates^18,19^.

While there are many similarities to organo-polyelectrolytes, metallo-polyelectrolytes provide a different level of electrolyte chemistry and physics. Noticeable difference is the presence of transition metals that allow unique electronic properties such as lipophobicity, ionic binding strength and redox chemistry^20,21^. The tunable oxidation state of metal cations would offer unprecedented redox chemistry that organo-ions typically cannot. This property could open up many new applications for metallo-polyelectrolytes beyond traditional organo-polyelectrolytes^8,22^. In addition, metal cations may exhibit significantly distinct behaviors in thermal and chemical stability for some applications^23,24^. On the other hand, metals are generally used as cationic centers for building polyelectrolytes, thus it is fair to compare metallo-polyelectrolytes with cationic organo-polyelectrolytes, particularly the most studied quaternary ammonium-based counterparts.

### Electronic properties of metallo-polyelectrolytes

The ionic binding for both metal-containing cations and organo-ions first encounters with electrostatic Coulombic interactions. The intrinsic electron configuration of transition metals often involves incompletely filled d-orbitals, which could have dramatically different electronic and possibly solvophilic effects in comparison with nitrogen and phosphorous atoms in organo-ions, when complexing with oppositely charged molecules. In the case of organometallic cations, the size of these cations matters. The Pearson theory of hard and soft acids and bases (HSAB theory) could provide a basic foundation to compare the ionic binding strength^25–27^. Strong binding occurs between hard acids and hard bases, similarly between soft acids and soft bases. Small quaternary ammonium is a hard acid and prefers to interact with small anions. In comparison, most metallo-polyelectrolytes carry large metal cations (or large organometallic cations), a kind of soft acids. Thus, they would favor to bind with soft bases, large anionic substrates^20^. Although there is lack of quantitative data on ionic binding strength in literature, the qualitative difference between organic and metal-containing ions should be considered for choosing counterions, whenever appropriate.

### Ionic binding of metallo-polyelectrolytes

**Fig. 3:**
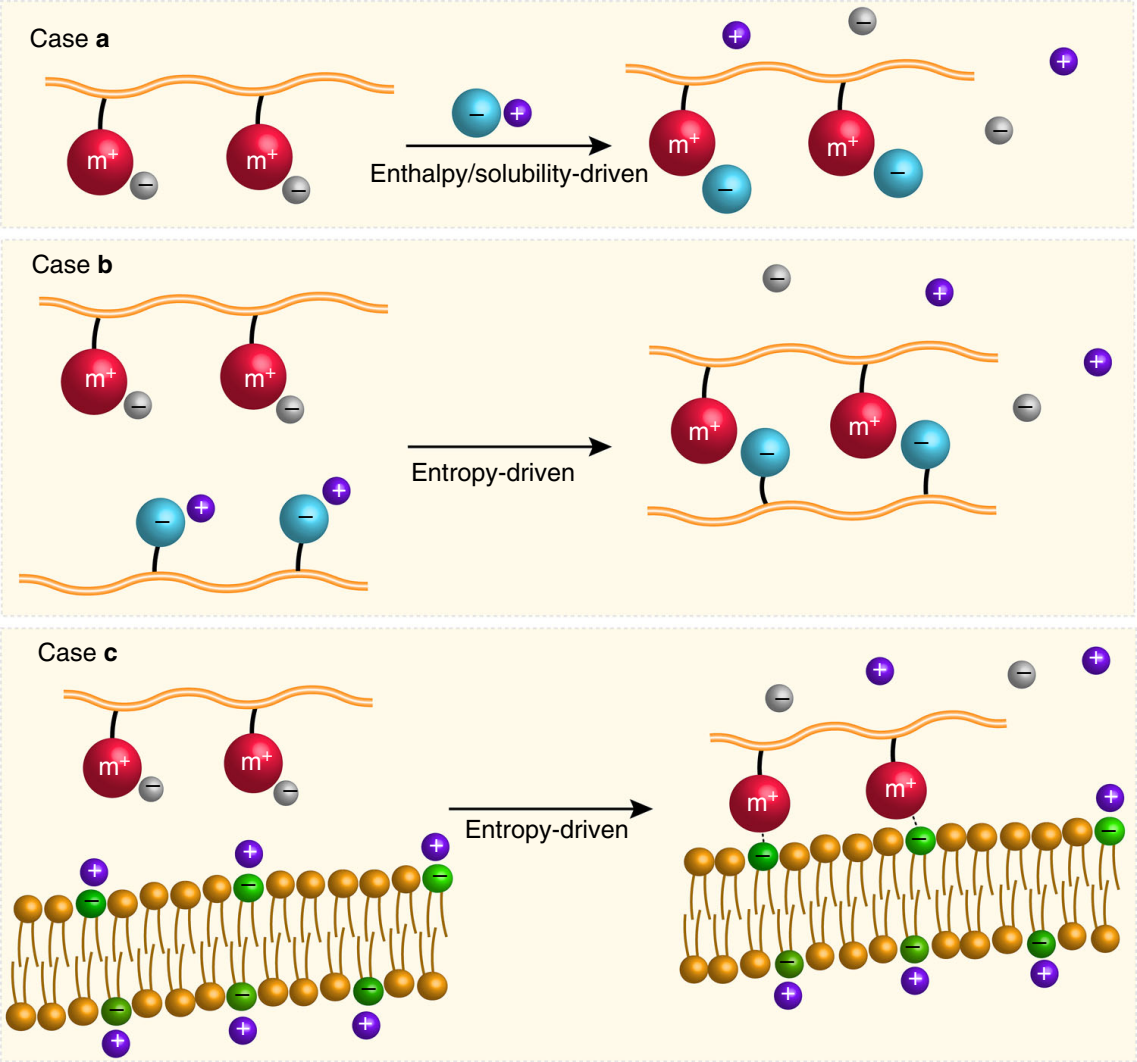
Metallo-polyelectrolyte interactions with oppositely charged molecular substrates at three different levels. **a** small molecules, **b** macromolecules, and **c** membranes or crosslinked networks

We examine interactions of metallo-polyelectrolytes with several oppositely charged substrates, which provide a general picture to illustrate various scenarios. Specific comparisons with organo-polyelectrolytes are given in following sections outlining functional materials. There are three levels of oppositely charged molecular substrates for interactions relevant to the functions of metallo-polyelectrolytes: small molecules, macromolecules, and membranes or crosslinked networks (Fig. 3). All these interactions are associated with changes of enthalpy, entropy, and in many cases dehydration (e.g. reduced solubility with loss of water).

In the case of molecular anions (Case a), the entropic change with the release of counterions associated with metal cations is generally small^28^. Thus, the enthalpic change would play a greater role in driving ion exchange. It has been demonstrated that the type of charges appears to be more important^29^. For example, carboxylates associate with cations weaker than sulfonates. The aromatic sulfonates associate more strongly than the aliphatic ones. The presence of π-system on anions increases the affinity for cationic metal centers significantly. This is mostly attributed to Coulombic interactions. Another driving force would be the reduction of solubility in solutions, in some case even leading to phase separation. The complexation of metallo-polyelectrolytes with molecular anions is essentially an equilibrium process, which could be dramatically shifted with the significant reduction of solubility or precipitation of newly formed complexes. For example, a phase-transfer ion-exchange strategy was developed to prepare cationic cobaltocenium polyelectrolytes using tetrabutyl ammonium halide salts to replace a hexafluorophosphate counterion^30^. The dramatically reduced solubility crashes out newly formed halide-paired polyelectrolytes in acetonitrile. On the other hand, halide-paired polyelectrolytes were reported to ion-exchange with antibiotics to form bioconjugates^31^. The exchange is favorable, possibly due to the small gain of entropy, as antibiotics have a significantly larger size than halides. In addition, one should consider the Pearson HSAB theory on soft acids and bases^25–27^. In the case of ion-exchange from chloride to β-lactam antibiotics, the anionic antibiotics are a relatively softer base than chloride, thus favorable for interaction with a soft acid like cobaltocenium.

When metallo-polyelectrolytes interact with negatively charged macromolecules, it is the situation of Case b for the formation of PECs. While the complexation starts with attractive electrostatic interactions, it is the favorable entropic change to drive the mixing by releasing their respective small counterions. The loss of configurational and translational entropy of the long chains of polyelectrolytes upon the formation of PECs would contribute positively to the entropy change. The PEC formation is dictated by many factors such as molecular weight, charge density, molar ratio, Van der Waals forces, hydrophobic interactions, hydrogen bonding, etc. Typically, polymers with a longer chain and higher charge densities tend to form PECs. It has been quantitatively compared with ionic binding strength between oppositely charged polyelectrolytes^29,32^. We reported the complexation of cobaltocenium-containing methacrylate polymers with poly(acrylic acid). The level of complexation was monitored by the release of their counterions. The gradual increase of turbidity indicated the formation of liquid-like coacervates^31^. However, when these metallo-polyelectrolytes interact with phosphate-containing macromolecules (e.g. lipoteichoic acid), it was observed with the formation of precipitates. In addition, when complexed with the same anionic polyelectrolytes (e.g. poly(styrene sulfonate)), cobaltocenium-containing polyelectrolytes exhibited stronger binding strength than quaternary ammonium-containing counterparts^20^.

The ionic binding strength can be evaluated by the extent to which ion pairs between PECs are torn apart by a common salt, KBr^29,33^. With the addition of salt, ion pairs in PECs are surrounded by the free ions via diffusion. With the increase of salt concentration, the PECs dissociate progressively. The complexation would be completely suppressed above a critical ionic strength, which depends on the type of ionic groups and charge density in polyelectrolytes as well as their chain length. Thus, ionic strength is always an important factor regarding polyelectrolyte complexation.

As a membrane or a crosslinked charged network possesses even lower translational and rotational configurations than a single polyelectrolyte (Case c), it is expected that the positive gain of entropy change would greatly favor its complexation with metallo-polyelectrolytes via releasing highly mobile counterions. On the other hand, the strong complexation might have a negative impact on the diffusion of polyelectrolytes across the network. The choice of ionic groups and charge density would become more important for tuning the complexation. It has been demonstrated that diffusion of ionic sites within PEC films is much faster than polyelectrolytes themselves (probably over 2 orders of magnitude)^34^. This is quite straightforward as it is a local rearrangement of ionic units vs. PEC assembly in all of their forms. Such diffusion becomes more relevant when the networks are cell membranes, which will be elaborated in a section below about antimicrobial biomaterials.

### Redox chemistry of metallo-polyelectrolytes

Research on redox active polyelectrolytes was actively explored in early 1980s^35–42^. For metallo-polyelectrolytes, metal-containing cations can be well utilized with their reduction and oxidation chemistry^43^, which is usually not possessed by organo-ions. For example, many research groups have extensively investigated the redox behaviors of ferrocenium and cobaltocenium-containing polyelectrolytes, including dendrimers and block copolymers^44–51^. The formation of PECs can be tuned by changing the oxidation state of metal-containing cations, in some cases, even fully destroyed. In the section below regarding multilayers, redox chemistry of metallo-polyelectrolytes could be utilized to modulate the assembly and disassembly processes. In biological systems, metal cations could participate metabolisms involving enzymes, such as oxidative stress^52,53^. Fenton chemistry is another common redox chemistry producing super radical species^54^, which has been reported in designing antimicrobial metallo-polyelectrolytes to induce oxidative stress on bacterial cells^55,56^, as elaborated below.

## Functional materials via redox or electrostatic interactions

### Polyelectrolyte multilayers (PEMs)

The formation of PECs is mostly exemplified by polyelectrolyte multilayers (PEMs), in which alternate polyanions and polycations are stacked on each other^57,58^. It is mostly an entropy-driven process. The loss of translational and configurational entropy is out-competed with entropic gains by releasing small counterions. Given diverse applications from functional coatings to advanced drug delivery vehicles, PEMs are typically constructed via strategies such as layer-by-layer, surface grafting, and Langmuir-Blodgett, which provide facile control over the surface characteristics and functionalities^59,60^. Different from organo-polyelectrolytes, the metal centers endow metallo-polyelectrolytes with tunable redox, photoluminescent, magnetic, and catalytic properties, and open up new horizons towards multifunctional smart PEMs^61–64^.

**Fig. 4:**
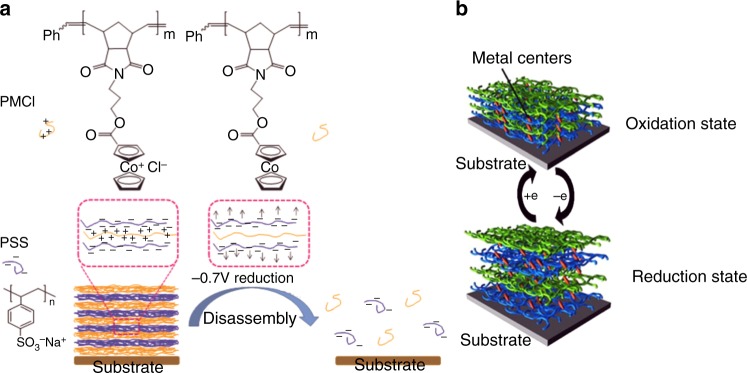
Controlled disassembly and controlled swelling of metallo-polyelectrolyte based PEMs. **a** reduction-induced disassembly of cobaltocenium-based PEMs^65^, adapted from ref.^65^ with permission from The Royal Society of Chemistry; **b** a strategy used in controlled swelling of PEMs^72^, adapted from ref.^72^ with permission, Copyright©2014, Elsevier

One of the key challenges in conventional PEMs lies in the controlled disassembly. Taking advantage of the redox-induced change of charged states of metal centers, many groups reported electrically controlled disassembly of PEMs based on metallo-polyelectrolytes^65–67^. It was found that PEMs constructed from a positively charged cobaltocenium-containing polyelectrolyte (PMCl) and negatively charged poly(styrene sulfonate) (PSS) underwent controlled disassembly upon electrical reduction of cobaltocenium^65^. This process is almost linearly dependent on the electrical reduction time, resulting in complete disintegration of PEMs in several minutes. This kind of electroactive PEMs was further explored as a trigger to control the release of non-responsive molecules in an overlaying layer. As shown in Fig. 4a, PEMs were assembled with the following compositions and sequence: PMCl(PSS/PMCl)(PSS/PDDA)_3_. Reduction of cobaltocenium caused a rapid and complete loss of the layers on top of PMCl(PSS/PMCl). Owing to the mild and controlled reduction of cobaltocenium, such kind of PEMs can be potentially used as effective triggers for redox-controlled release of analytes that are not responsive and may be sensitive to harsh redox conditions, such as drugs and antibodies.

How to control mechanical properties and swellability of PEMs is critical for many applications such as tissue engineering and drug delivery^21,68^. In theory, any influxes that contribute to the osmotic pressure and the electrostatic repulsion of likely charged polyelectrolytes may cause PEMs to swell. In the case of metallo-polyelectrolytes, once the redox state of metal centers is changed, corresponding counterions have to diffuse out of the multilayers to compensate for the missing charges. As a result of the increase in osmotic pressure, the PEMs start to swell due to their elastic polymer deformations^69^. Different redox-active metal centers involving ferrocene, Os(bpy)_2_PyCl^+^, or Os(CN)_6_Py^3−/2−^ have been used in electrochemical swelling of PEMs^21,70,71^. Calvo et al. reported the first example of electrochemical swelling of PEMs^70^, as shown in Fig. 4b^72^. They used osmium complex–containing PAH (poly(allylaminehydrochloride)) and glucoseoxidase (GOx) or PSS to assemble PEM films. It was found that during the oxidation/reduction of osmium complexes, the PEMs underwent reversible swelling and deswelling. It was attributed to the incorporation or exclusion of counterions and/or water molecules from the electrolytic solution^70^.

The presence of metal centers in metallo-polyelectrolytes could endow PEMs with catalytic properties. There are two major approaches to fabricating this kind of PEMs. One approach is to install catalytic sites in PEMs by using precursor-containing polyelectrolytes. Rahim et al. used a polyelectrolyte complex of silver ion-doped polyethyleneimine (PEI) and poly(acrylic acid) (PAA) to fabricate PEMs^73^. After in-situ reduction of Ag^+^ to Ag nanoparticle, it was found that the hybrid films exhibited an enhanced catalytic activity on the reduction of 4-nitrophenol. The other method uses charged coordination supramolecular polyelectrolytes. For example, Liang et al. prepared PEMs with dual electrocatalytic activity by using supramolecular metallo-polyelectrolytes carrying Fe^3+^ and a bis-ligand^74^. These films may lower the half-wave potential for different kinds of electroactive molecules and enhance the current for neutral or oppositely charged electroactive species, which are attributed to the mediation of metallo-polyelectrolytes in the electron transfer process and the accumulation of electrons in the films. The thickness of PEMs is very crucial in this case, which may affect the depth where the exchange of electrons occurs. If the thickness is less than 25 nm, there are some uncovered sites on the electrode, therefore, the electroactive species can diffuse quickly to the exposed electrode surface before electron exchange occurs. While in the case of a thicker film, the electrode is covered completely, which prevents the diffusion of electroactive species. Miao and coworkers prepared side-chain metal cluster-based polymers through ring-opening metathesis polymerization^75^. The metal centers in the polyoxometalate cluster are at their highest charged state, which makes the cluster a potential catalyst for oxidation. By using a model oxidation reaction of tetrahydrothiophene, they compared the catalytic property of both monomer and polyelectrolyte. Quantitative experiments demonstrated that the polymer showed a better catalytic activity than its corresponding monomer.

PEMs from metallo-polyelectrolytes, especially metallo-supramolecular coordination polyelectrolytes (MEPEs)^76,77^, demonstrate reversible electrochromism with applications including dynamic windows, panel displays and smart paper. Most MEPEs are constructed by ditopic ligands, such as bis-terpyridine and its analogues, and transition metal ions (Fe^2+^, Co^2+^, Ni^2+^, Zn^2+^, Ru^2+^, etc). Typically, the electrochromic properties of this system are related to the redox-dependent metal-to-ligand charge transfer (MLCT)^78^, as well as the nature of substituents and spacers^79^. For example, the Kurth group reported a series of MEPEs with bis-terpyridines with different conjugated spacers^80^. They found that the length of spacers has an influence on the color of PEM films. Upon the increase of electrical potential from 0 to +1.6 V (vs. Ag/AgCl), the absorbance of MLCT at ca. 585 nm decreased, and the color of films decayed from dark-blue to colorless. Full recovery of MLCT can be achieved by decreasing the potential back to 0 V. The electrochromism was almost fully reversible over 150 cycles. In another case, by using cationic viologen group as a spacer, Zhang and coworkers prepared electrochromic PEMs using a Langmuir-Blodgett method for alternate deposition of MEPE and PSS^79^. Upon the reduction of ligand with an applied potential of −1.1 V vs. Ag/AgCl, films changed from colorless to light red, which was also reversible after removal of the applied potential (−1.1 V).

Compared with organo-polyelectrolytes, magnetic properties are another unique characteristic for metallo-polyelectrolytes. By carefully selecting metal ions and ligands, magnetic metallo-polyelectrolytes could have tunable magnetic properties^81–85^. Owing to the decrease of the crystal field splitting of d-orbital subsets, metal ions in metallo-polyelectrolytes may undergo a spin transition from a low-spin to a high-spin state, resulting in changes of magnetic properties. By combining ditopic ligand and Fe^2+^, the Kurth group reported a metallo-supramolecular coordination polyelectrolyte wrapped with flexible alkyl chains, which are electrostatically encapsulated with flexible quaternized surfactants^82^. Subsequently followed by a Langmuir–Blodgett technique, multilayers were prepared. The hexagonally packed alkyl chains became disordered upon heating, resulting in a distortion of the metal ion coordination geometry. The changes in the coordination geometry of the metal complex resulted in a reversible spin crossover (SCO) between a diamagnetic low-spin state and a paramagnetic high-spin state. Besides heat-induced SCO, Yan and coworkers also reported a redox-induced SCO system based on a polyelectrolyte complex between a cationic organo-polyelectrolyte and a coordination metallo-polyelectrolyte from ditopic ligand and Fe^2+^^83^. Upon oxidation to Fe^3+^, the polyelectrolyte complex became paramagnetic due to the presence of an odd number of unpaired electrons, which resulted in a decrease of both the spin–lattice relaxation time *T*_1_ and the spin–spin relaxation time *T*_2_ of protons in water.

Taking the advantage of UV absorption ability of metallo-polyelectrolytes and its conversion to heat, Burnworth and coworkers reported an optically healable supramolecular metallo-polyelectrolyte^86^. On exposure to ultraviolet light, the metal complexes can be electronically excited, and the absorbed energy is converted into heat. This causes temporary disengagement of the metal-ligand motifs and subsequent reversible decrease in the molecular weight and viscosity of the polyelectrolyte, thereby allowing quick and efficient healing of defects. The photoluminescent properties of metallo-polyelectrolytes can be also used in label-free detection of proteins. For example, a cationic platinum-terpyridine complex and anionic poly(phenylene ethynylene) with sulfonate groups in the side chain (PPE-SO_3_^−^) were used to form a polyelectrolyte complex^87,88^. They found Fӧrster resonance energy transfer (FRET) from the polymer backbone to the metal complex can be tuned upon interactions with biomacromolecules, which can be used to detect human serum albumin.

Besides PEMs, another method to construct surfaces with metallo-polyelectrolytes is self-assembled monolayers (SAMs)^89^, which allow precise control of location and morphology. Traditional SAMs are usually fabricated from functional thiols and silanes. While in the case of metallo-polyelectrolytes, the Geiger group developed a covalent modification method through cathodic reduction of cyclopentadienyldiazonium complexes^90^. Cobaltocenium with diazonium groups underwent facile cyclopentadienyldiazonium ligand-based one-electron reduction on the surface of a glassy carbon electrode. Accompanied by the release of nitrogen gas, a metal-containing monolayer was installed on the electrode. Due to the nature of covalent bonding, these monolayers are quite robust with long-time storage. Recently, they also achieved anodic oxidation of cobaltocenium monolayer-modified electrodes^91^. By incorporating metallo-polyelectrolytes or metal precursors, the swelling of SAMs can be tuned as a function of electrode potentials^92^.

### Antimicrobial biomaterials: ionic interactions with cells

Via enthalpy or entropy or both driven ion exchange, metallo-polyelectrolytes can bind to small anionic molecules like antibiotics, negatively charged macromolecules like peptides, enzymes and proteins^32,93^, as well as anionic networks like cell walls and membranes^94^. Taken together with the redox metal centers, metallo-polyelectrolytes can facilitate intracellular biochemical processes, thus providing unprecedented opportunities for antimicrobial applications.

Drug resistance has become an increasingly intractable issue that leads humanity towards a post-antibiotic era^95^. The discovery of next-generation antimicrobial agents is aimed to address this mounting crisis. As a comparison to small molecular antibiotics, antimicrobial macromolecules are usually more chemically robust, less volatile, with longer lifetime of killing efficacy and superior environmental compatibility^56,96–99^. Among them, metallo-polyelectrolytes show some of unique characteristic as they integrate multiple antimicrobial moieties into a macromolecular scaffold. Currently, various types of metals, including iron, cobalt, tin, copper, nickel, ruthenium, platinum, gold, silver, zirconium, titanium, vanadium, chromium, cadmium, manganese, zinc, germanium gallium and lanthanum, have been integrated into macromolecular architectures to achieve antimicrobial properties^8,56,100,101^. Among them, iron, cobalt, ruthenium, copper and silver are mostly explored. There are two major types of antimicrobial mechanisms of action by metallo-polyelectrolytes: (1) metal ions provide the antimicrobial activity; (2) metallo-polyelectrolyte bioconjugates with antibiotics enhance antimicrobial activity.

**Fig. 5:**
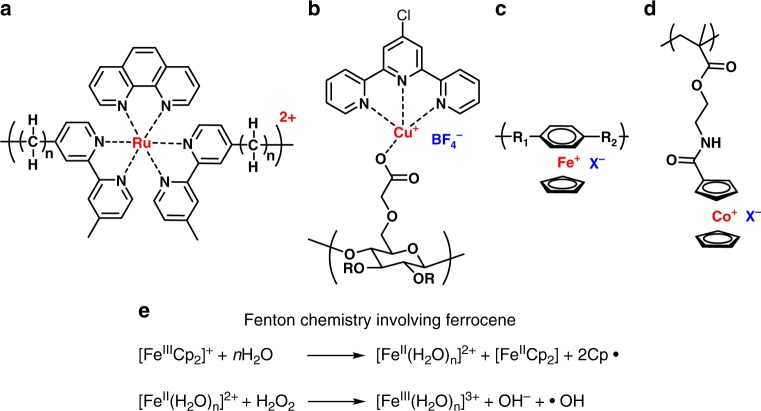
Structural units of antimicrobial metallo-polyelectrolytes. **a** polypyridylruthenium(II) complex polymer;^102–104^ **b** copper-terpyridine carboxymethyl cellulose polymer;^105^ **c** η^6^-arene-η^5^-cyclopentadienyliron(II) complex polymer;^107–110^ **d** cobaltocenium polymer;^117^ **e** Fenton chemistry involving redox of ferrocene^111^

In the first case, metal cations can directly chelate with various intracellular biomacromolecules like enzymes and proteins to modulate biochemical processes that mediate antimicrobial behaviors. Metal cations have been incorporated into main chain or side chain macromolecular frameworks, which can be transported to specific cellular environments. For example, ruthenium ion can form a complex by coordinating with pyridine through a flexible polymethylene linker to yield ruthenium-based main-chain polyelectrolytes (Fig. 5a). The Keene group reported multi-nuclear polypyridyl ruthenium(II) complexes as antimicrobial agents that can accumulate on bacterial cells. The ruthenium complexes interfere with key cellular processes to kill bacteria^102–104^. Hassan et al. reported a side-chain water-soluble cupric cellulose hybrid via a supramolecular strategy with tetrafluoroborate as the counter anion (Fig. 5b)^105^. This copper-based polyelectrolyte greatly inhibited the growth of Gram-negative *E. coli* and Gram-positive *S. thermophiles* and *S. aureus* by blocking metal-binding sites in enzymes and disturbing the cell respiration process. In addition to specific intracellular biomedical processes involving metal-ligand binding, the tunable oxidation state of metal cations would offer unique redox chemistry that enables Fenton-type reaction to trigger cellular oxidative stress on bacteria^55,106^. Iron-based polyelectrolytes utilize a cationic derivative of ferrocene, termed η^6^-arene-η^5^-cyclopentadienyl iron(II) complex (Fig. 5c), reported by Abd-El-Aziz and coworkers^107–110^. The iron complex-containing linear polymers and dendrimers can induce cellular oxidative stress on bacteria due to the redox-active cationic iron center. A simple illustration of Fenton chemistry of ferrocene is given in Fig. 5e^111,112^. In addition, the antimicrobial activity can be easily tuned by changing the counter anion^108^.

Given the cationic nature of metallo-polyelectrolytes, they could ion-pair with anionic peptides to form bioconjugates, which have shown some of unique antimicrobial efficacy, especially towards drug-resistant bacteria, in comparison to unconjugated polyelectrolytes. Joyner et al.^113^ reported that metallo-peptides containing cupric ion complexes showed more enhanced antimicrobial activities than each structural unit alone. They claimed that metal ion-containing peptides irreversibly cleaved cellular nuclei acids. In addition, the charged metal complexes can promote oxidation with proteins, redox cofactors, or initiate a cellular oxidative stress response that ultimately damaged bacterial cells. In addition to cupric ions, cationic cobaltocenium ions can also bind with peptides. Metzler-Nolte and coworkers reported a series of cobaltocenium-containing polypeptides. The antimicrobial activities towards Gram-positive *S. aureus* and Gram-negative *E. coli* and *P. aeruginosa* were enhanced due to the increased cationic charge units from cobaltocenium, which strengthened the electrostatic binding to negatively charged bacterial membranes^114–116^.

**Fig. 6:**
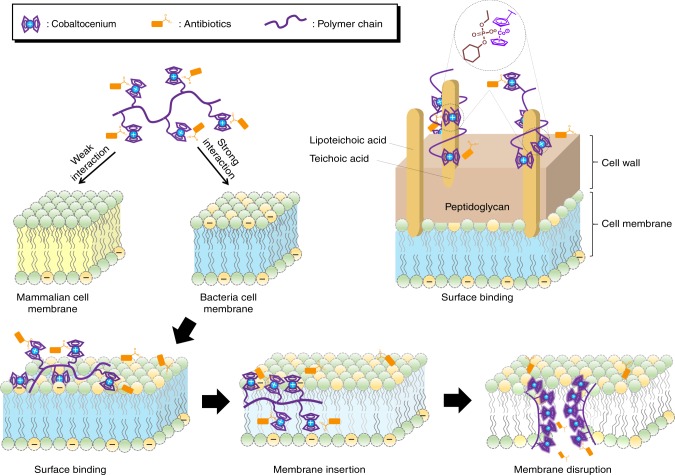
Proposed interactions between metallo-polyelectrolytes and bacterial cells. It involves the following steps: electrostatic interactions between cationic metallo-polyelectrolytes and anionic membranes; surface binding on membranes, membrane insertion and eventual membrane disruption. Metallo-polyelectrolytes could also interact with lipoteichoic acid in the outer leaflet of Gram-positive bacteria

In addition to peptides, the affinity of cationic cobaltocenium species with anionic substrates enables them to form bioconjugates with many traditional antibiotics via electrostatic binding. Recently, the Tang group reported cobaltocenium-containing polymers as metallo-polyelectrolytes for antimicrobials^31,117,118^, as shown in Fig. 5d. These metallo-polyelectrolytes can conjugate with widely used β-lactam antibiotics such as penicillin-G, amoxicillin and ampicillin, providing a unique macromolecular scaffold to revitalize traditional antibiotics to kill multidrug-resistant bacteria. As indicated in Fig. 6, antibiotic-loading quantities could be mediated by the cobaltocenium moiety on a polyelectrolyte chain. When cobaltocenium polyelectrolytes come closer to the crosslinked cell wall, the electrostatic interactions with negative lipoteichoic acid facilitate binding of metallo-polyelectrolytes with microbial cells. Alternatively, cationic cobaltocenium polyelectrolytes can strongly interact with anionic phospholipid on microbial cell membranes. These processes are possibly driven by the increase of entropy via releasing small antibiotics. The amphiphilic nature of polyelectrolytes would also promote insertion into membranes, leading to their disruption^119^.

At the same time, cobaltocenium polyelectrolytes also act as β-lactamase inhibitors. β-Lactamase is an enzyme, which contains negative carboxyl groups in a few amino acid residuals. The PEC-like binding between metallo-polyelectrolytes and β-lactamase is an entropy-favorable process by releasing small counterions, effectively nullifying the activity of β-lactamase^29^. As observed by Lindhoud et al.^120^, a similar inhibition behavior of enzymatic activity occurred when they added more positively charged units onto polyelectrolytes. Therefore, cobaltocenium polyelectrolytes have shown synergistic effects to lyse drug-resistant bacterial cells whereas being nontoxic towards mammalian cells due to much weaker hydrophobic-hydrophobic interactions^31,117^. In addition, attempts were made to increase the antimicrobial activity in a more refined manner via structural modification. For example, boronic acid was introduced into a metallo-polyelectrolyte framework to make it adhering to lipopolysaccharide or peptidoglycan to further enhance interactions with cells^117^. Cobaltocenium-containing polyelectrolytes were also grafted from inorganic nanoparticles such as silica and iron oxide to increase the local drug concentration due to their high specific surface area^121,122^. Owing to the facile synthetic methodology and diverse well-established antibiotic counterparts, metallo-polyelectrolytes establish a platform of therapeutics against bacteria.

Organo-polyelectrolytes contain quaternary ammonium, phosphonium or sulfonium groups as the cationic centers in comparison to metallo-polyelectrolytes. The cationic groups can selectively bind to bacterial membranes via electrostatic interactions, subsequently hydrophobic components penetrate into the lipid layer, resulting in cell membrane disruption and eventually cell death. Metallo-polyelectrolytes could perform similar mechanisms of action, but much more beyond organo-polyelectrolytes. Metal cations can directly chelate with various intracellular biomacromolecules like enzymes or proteins whereas traditional organo-polyelectrolyte may not. When organo-polyelectrolytes are too hydrophilic, they are inactive in disrupting bacterial membranes. However, when the hydrophobicity is too high, they could be toxic towards mammalian cells, as driven by hydrophobic interactions with zwitterionic mammalian cell membranes. Therefore, the antimicrobial activity is achieved by the optimal compositions of organo-polyelectrolytes, sometimes the counterbalance between binding selectivity and membrane disruption can be a dilemma. In the case of the formation of bioconjugates, metallo-polyelectrolytes can circumvent such problem because only clinically approved antibiotics or peptides play a role in killing bacteria, while the metallo-polyelectrolyte framework facilitates their revitalization of efficacy by nullifying bacterial resistance pathways.

### Gene delivery of polyplexes

As mentioned above, metallo-polyelectrolytes can form PECs with oppositely charged polyelectrolytes. When they interact with negatively charged biomacromolecules like protein, DNA and enzymes, they can form a complexation favorably by releasing small counterions. The state of PECs could be either liquid-like coacervates or solid precipitates, depending on the strength of complexation. The binding ability of cationic metallo-polyelectrolytes with anionic biomacromolecules could provide opportunities for gene delivery.

Nucleic acid-based therapeutics have gained extensive attention over the past two decades due to their promise for the treatment of many life-threatening diseases, ranging from genetic disorders to cancers^123,124^. Successful delivery of nucleic acids depends on the design of safe and efficient vectors^3^. There are two major delivery vectors to transport nucleic acid-based therapeutics including plasmid DNA and small interfering RNA (SiRNA): viral and non-viral vectors^125^. Non-viral vectors, especially synthetic cationic macromolecules, have precisely customized chemical structures and functionalities, leading to a great improvement in cost and safety compared to viral vectors^126^.

Via ionic interactions, a cationic polyelectrolyte can bind with DNA to form a polyplex, a terminology essentially same as PEC. The mostly explored polyelectrolytes are poly(L-lysine) and polyethylenimine as well as their modified variants^125^. Despite extensive research on these traditional organic amino-polyelectrolytes, examples of other polyelectrolytes show their burgeoning opportunities as alternatives^127,128^. To the best of our knowledge, the only reported metallo-polyelectrolytes with charged metal centers can be potentially used for gene delivery are cobaltocenium bioconjugates, as reported by Metzler-Nolte and coworkers^115^. They found that cobaltocenium-peptide bioconjugates exhibited active endocytosis and directed nuclear delivery, which are two key steps for gene release. Manners et al.^129,130^ prepared a main-chain cobaltocenium-containing water-soluble polyelectrolyte termed poly(cobaltoceniumethylene). Cationic cobaltocenium ions can induce conformational replications of an anionic DNA template through electrostatic interaction, demonstrating the efficient formation of polyplexes^128,131^. These efforts could inspire the use of cationic metallopolymers as gene delivery vehicles, as the formation of polyplexes would be the first step to carry genetic macromolecules.

**Fig. 7:**
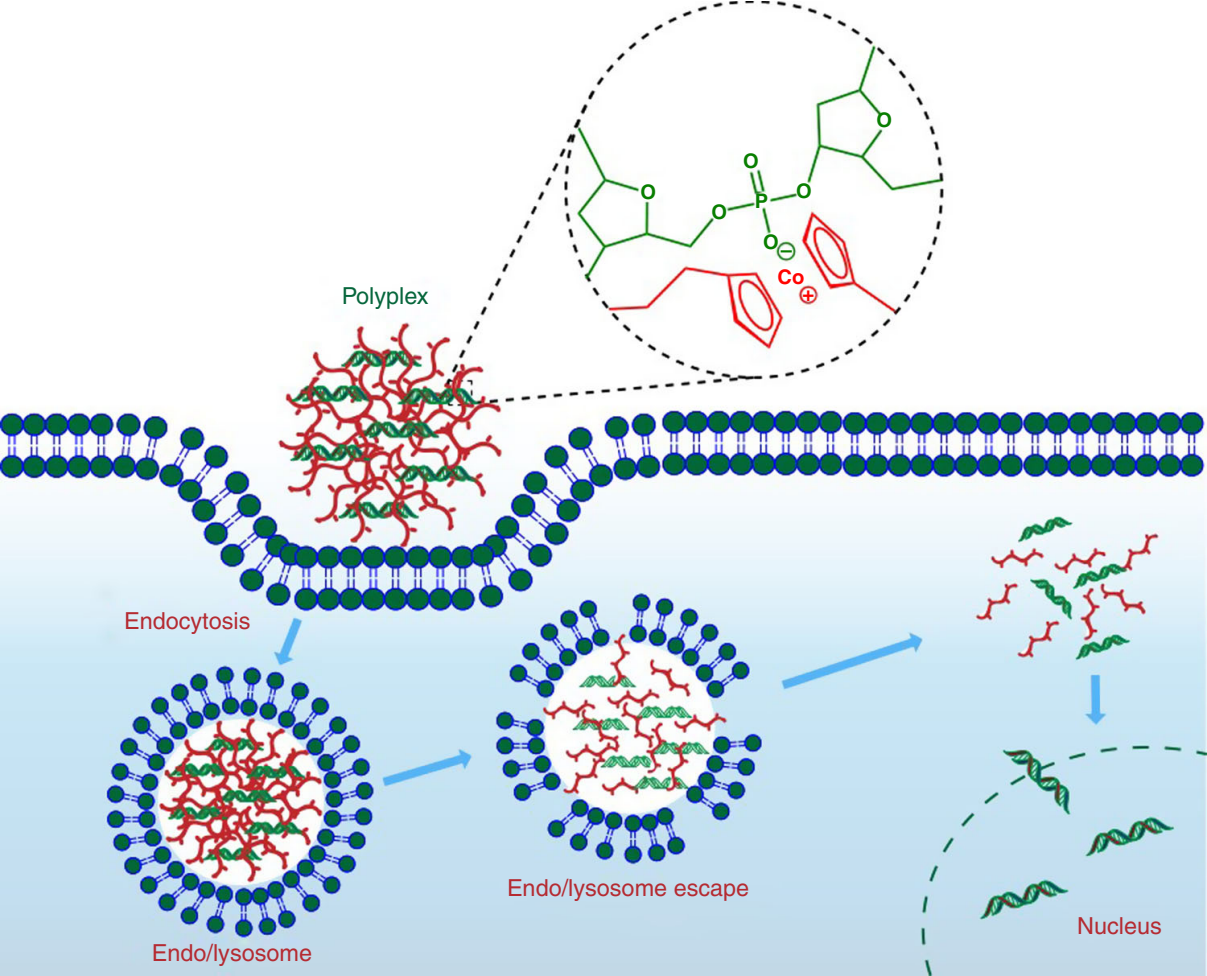
Proposed dene delivery of non-viral metallo-polyelectrolytes. The delivery requires the formation of polyplexes between metallo-polyelectrolytes and nucleic acids. It involves endocytosis, endo/lysosome escape and release of nucleic acids

A possible delivery of cobaltocenium polyelectrolyte-DNA polyplexes is proposed in Fig. 7. Polyelectrolytes with positively charged cobaltocenium can strongly bind with the anionic phosphodiester group of a nucleic acid to form colloidally stable polyplexes. Polyplexes enter cells via endocytosis and are trapped in endosomes. Then, rupture of endosomes and release of DNA to cytosol may occur through a proton sponge effect (pH-buffering)^125^. After an active endocytosis by cells, it forms an endo/lysosome. Then the acidic endo/lysosome microenvironment can hydrolyze the ester linkers in cobaltocenium copolymers, the membrane is disrupted by the hydrophobic chain segments, facilitating the release of DNA. Eventually, DNA chains penetrate into nucleus for gene therapy^132^.

As a comparison to metallo-polyelectrolytes with charged metal centers, a polyelectrolyte with neutral metal centers used in gene delivery contains ferrocene as the organometallic moiety^127^. The charged centers are quaternary ammonium, which binds with the anionic DNA chain. The ferrocene moiety along the polyelectrolyte backbone can play a hydrophobic role or redox-active activity rather than electrostatic interaction^133,134^.

## Ion-exchange for transport

Ion-exchange in polyelectrolyte-based membranes has a broad range of applications in energy conversion/storage devices, such as alkaline fuel cells^135,136^, redox flow batteries^137^, electrodialysis and electrolyzers^138^. It mainly takes the advantage of the ability of binding to small ions (like hydroxide, halide, carbonate ions) and redox stability under operational potentials. However, it remains a challenge to develop advanced anion-exchange membranes (AEMs) with sufficient chemical stability and high anionic conductivity under extremely harsh, highly basic conditions in alkaline fuel cells. The major issues that limit the performance of AEMs include the degradation of polymers under caustic conditions and lower intrinsic mobility of hydroxide ions. This section summarizes recent progress on the design of metallo-polyelectrolytes to improve ion exchange as well as cation stability under alkaline conditions.

Traditional quaternary ammonium-based organo-polyelectrolytes are used for anion-exchange and transport^139–141^. Some other organic cations include imidazolium^142,143^, phosphonium^144,145^, pyridinium^146^, and guanidinium^147,148^, while poor chemical and thermal stability is the major concern for many of these cations and their organo-polyelectrolytes. The primary mechanisms of degradation for the well-studied benzyl trimethylammonium cation involve Hofmann elimination and nucleophilic substitution by hydroxide ions, as well as the formation of Ylide or chemical rearrangements^136,149^.

Compared to conventional organic cations, many metal complexes (or metal cations) exhibit good physiochemical stability under strong basic conditions. For example, cationic Ru complexes exhibited excellent stability in NaOH solution at room temperature over six months^23^. Similarly, no significant changes were observed for cobaltocenium cyclooctene monomer in 1 M NaOH solution at 80 °C for two weeks^24^. Only 7% of dimethyl cobaltocenium cation degraded after storing in 1 M KOH at 80 °C for 30 days^150^. Permethyl cobaltocenium cation showed a much longer lifetime than many other organic cations under high temperature (only 8.5% degraded at 140 °C in 1 M NaOD/D_2_O after 6 weeks)^151^. In addition, both mono-substituted and permethyl cobaltocenium-containing polyelectrolytes showed excellent thermal stability with decomposition temperature over 300 °C, which is significantly higher than most ammonium or imidazole-functionalized polyelectrolytes^24,151^. However, more rigorous tests are required to reveal the stability of these metal complexes under highly basic and also highly oxidative conditions, especially at the device level^152^. In addition to metal-containing ions, polymeric frameworks are equally important to maintain structural and mechanical integrity of the entire polyelectrolytes.

**Fig. 8:**
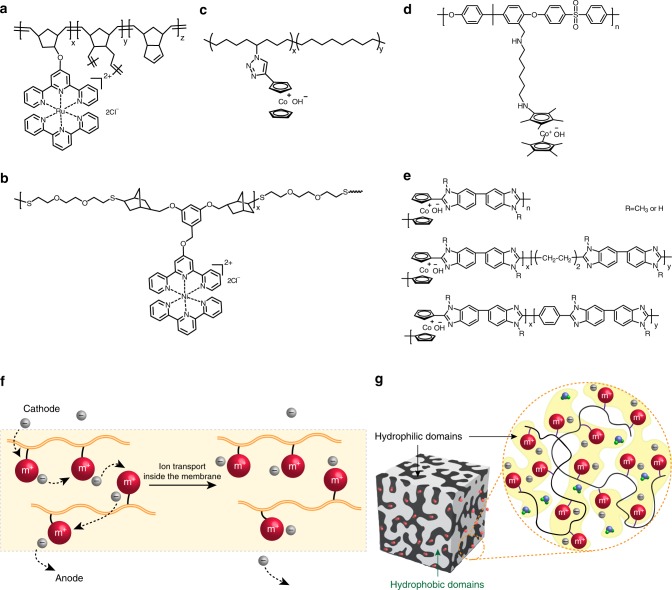
Metallo-polyelectrolytes for ion transport. Synthesis of metallo-polyelectrolytes by ROMP with metal complexes at the side-chain: **a** heteroleptic bis(terpyridine) Ru(II) complex;^23^ **b** nickel-containing complex;^154^ **c** cobaltocenium cation^24^. Metallo-polyelectrolytes by polycondensation: **d** permethyl cobaltocenium-containing polysulfone;^151^ **e** cobaltocenium-containing polybenzimidazole;^150^ **f** illustration of single ion site migration inside alkaline anion-exchange membranes; **g** A highly preferred phase separated morphology containing continuous hydrophilic ion transport channels and a hydrophobic matrix^155^, adapted with permission from ref.^155^. Copyright © 2017, American Chemical Society

On the other hand, to obtain higher ion conductivity, elucidation of the ion diffusion mechanisms inside polyelectrolyte membranes is vital^34,153^. In the case of alkaline fuel cells, free hydroxide ions are produced by oxygen reduction reaction in the cathode. Then, hydroxide ions can hop from one cationic site to another, as seen in Fig. 8f, which is similar to proton transport through the ionic domains in Nafion. Finally, free hydroxide ions transport through membranes to the anode side and participate the oxidation reaction with fuels. We reported cobaltocenium-containing copolymers with a hydrophobic polyethylene backbone for maintaining mechanical stability and hydrophilic cobaltocenium side chains for achieving rapid hydroxide transport^24^. To date, metallo-polyelectrolytes for ion-exchange membranes can be separated into two categories: polymers with non-aromatic based backbones and aromatic backbones.

### Non-aromatic based polymer backbones

This class of metallo-polyelectrolytes is mostly built from norbornene/cyclooctene-functionalized monomers, as shown in Fig. 8a–c. Hickner, Tew and co-workers reported multivalent metal-cation-based polyelectrolytes in 2012^23^, which were synthesized by copolymerization of a bis(terpyridine)Ru(II) complex-functionalized norbornene monomer with dicyclopentadiene, the latter monomer is a crosslinker (Fig. 8a). These membranes exhibited good hydroxide conductivity (28.6 mS/cm at 30 °C) with mechanical properties comparable to traditional quaternary ammonium-based polyelectrolytes. In early 2018, they further developed crosslinked nickel-containing polyelectrolytes using thiol-ene reactions (Fig. 8b)^154^. In this case, the nickel cation functions as both an ion conductor and a crosslinker. Although the formation of ion clusters was observed, high water uptake ( > 150%) and extreme brittleness of membranes were the major drawbacks. We reported ring-opening metathesis polymerization (ROMP) of cobaltocenium-containing cyclooctene, followed by backbone hydrogenation, leading to a class of membranes with a polyethylene-like framework and alkaline-stable cobaltocenium cation (Fig. 8c)^24^. These polyelectrolyte membranes showed high hydroxide conductivity (90 mS/cm at 90 °C) and excellent durability over one month.

### Aromatic based polymer backbones

The second type of metallo-polyelectrolytes contains aromatic polymer backbones. These polymers are typically prepared by polycondensation reactions. The Yan group reported permethyl cobaltocenium as an ultra-stable cation for ion transport^151^. The bulky hydrophilic cations were linked to a rigid hydrophobic polysulfone backbone via a flexible diamine linker (Fig. 8d). The membrane exhibited excellent thermal stability, high tensile strength (40 MPa with 10% elongation at break) and good conductivity (22 mS/cm at room temperature). However, earlier work demonstrated that polysulfone-based membranes would have cation-triggered backbone degradation^149^, which remains a concern for durability of these materials. Polybenzimidazole membranes with cobaltocenium on the backbone were prepared by microwave polycondensation, as reported by Zhu and co-workers (Fig. 8e)^150^. These membranes exhibited hydroxide conductivity of 37.5 mS/cm at 90 °C and over 80% hydroxide conductivity retention for nearly one month.

Ideally, a phase-separated morphology in membranes is favorable to facilitate the ion transport and improve the electrochemical performance, especially under partially hydrated conditions, as illustrated in Fig. 8g^155^. Well-connected hydrophilic domains or nanochannels for faster ion migration were also observed in many polymer architectures like block copolymers and comb-shaped polymers^156^. On the other hand, hydrophobic components could serve as the matrix to maintain structural and mechanical integrity during ion transport and device fabrication.

## Conclusions and perspectives

The field of metallo-polyelectrolytes is just beginning to take off, as a result of their fascinating electronic, physicochemical and redox properties that are recently discovered as quite differently from organo-polyelectrolyte counterparts. The unique electronic properties of metal centers endow metallo-polyelectrolytes some of unprecedented ionic binding with a variety of (macro)molecular substrates. They could complex with small antibiotics, oppositely charged polyelectrolytes such as DNA and peptides, as well as cell membranes or crosslinked networks. The redox properties further allow metallo-polyelectrolytes to access many functions that traditional organo-polyelectrolytes cannot. Metal cations also possess unusual physiochemical stability, which could be well utilized in applications requiring robust ion exchange and transport. Given these rich properties, we summarize below specific applications and challenges, and provide future perspectives of metallo-polyelectrolytes.

The application of metallo-polyelectrolytes for polyelectrolyte multilayers not only enables emerging methods of assembly, but also introduces multiple functions and responsiveness. With the abundant choices of transition metal elements from the Periodic Table, it is believed that metallo-polyelectrolyte-based PEMs could find more applications from functional coatings to biomedical utilities. The redox properties of metals allow controlled disassembly, swelling, electrochromism, etc. On the other hand, metallo-polyelectrolytes have shown some superior antimicrobial activities due to synergistic mechanisms of action, which combine the intrinsic ability of metal ions and unexpected efficiency of ionic complexation. Benefited from the versatile methodology of macromolecular synthesis, metal moieties can be conjugated into polymeric scaffolds in a more refined manner. It is envisioned that metallo-polyelectrolytes have great potential in fighting against multi-drug resistant bacteria. Metallo-polyelectrolytes can be also a promising non-viral vector candidate for efficient gene delivery, though most research is still at the early infancy stage. Many traditional amino-based polymers suffer from significant toxicity and instability of polyplexes with nucleic acids under physiological environments. In comparison, a few metallo-polyelectrolytes exhibit robust chemical stability toward harsh conditions and nontoxicity for mammalian cells. Finally, cationic metallo-polyelectrolytes have been utilized for ion exchange and transport, particularly for hydroxide ions in electrochemical devices. They are promising materials for the next-generation anion-exchange membranes. There have been no reports on cell performance on these metal-containing membranes yet. Further improvement on alkaline and chemical stability and ionic conductivity is needed. A highly stable metal cation is necessary, but insufficient. More stable polymer backbones and linkages are also essential.

However, there exists significant challenges and hurdles facing metallo-polyelectrolytes, which need to be addressed in order to achieve greater emerging properties and applications:

Quantitative analysis of ionic binding strength of metallo-polyelectrolytes with molecular substrates is largely missing. While similar dilemma is faced with the entire field of polyelectrolytes, our understanding on metallo-polyelectrolytes is almost exclusively qualitative. For organo-polyelectrolytes, quantitative interaction strength of PECs has been directly probed by the ability of salts added to break ion pairs using spectroscopic methods. The Schlenoff group have pioneered on the salt-water plasticization of PECs (“saloplasticity”) to extrude dense shapes suitable for many types of measurements, including ion equilibria^29,157^. However, quantitative binding with small molecules or membranes is critically needed. An establishment of representative phase diagram would be a challenging, but worthy task to explore^158^. There is an urgent need to establish quantitative physical chemistry of metallo-polyelectrolytes.

Theoretical models are needed to construct and predict key influential parameters on polyelectrolyte complexation^28,159^. These models could combine with experimental analysis to augment the understanding at molecular and macroscopic levels. A mean-field model of complex coacervation from weakly charged polymers has been established by Voorn and Overbeek^160^. Parameters include chain length, charge density and concentrations of polymers and salts. Random phase approximation allows high charge densities and connectivity of polymer segments, as studied in early 2000^161,162^. Later, an off-lattice approach describing polyelectrolyte complexation for polyelectrolytes with pH-dependent charges was constructed by Biesheuvel and Cohen Stuart^163^. Very recently, a coarse-grained model is proposed to simulate coacervation as a function of polymer length and overall salt concentration by the de Palbo group^164^. These models could help estimate the total free energy of mixing as well as mixing entropy and enthalpy terms. However, these models only deal with organo-polyelectrolytes. New models or advanced versions of the above models must take consideration of metal cations and their unique electronic and redox properties.

Grand challenges would be dealing with more complex biological interfaces. For example, multiple mechanisms of action have been proposed for antimicrobial activity of metallo-polyelectrolytes. However, the intrinsic free energy change such as enthalpy and entropy change as well as solubility equilibrium, which ultimately interferes key biochemical processes, is still not quantitively studied. A more comprehensive understanding is needed to direct the design of macromolecular metal complexes, i.e., macromolecular structures, complex types and metal moieties. Another example involves ion transport. Theoretical modelling could help design new metallo-polyelectrolytes with better phase-separated structures for improved electrochemical performance.

Nevertheless, the fields of polymer and macromolecular sciences have overseen the enormous potential of metallo-polyelectrolytes for a broad range of fundamental scientific mysteries and practical applications. Much work on the chemistry, biology, physics and engineering of metallo-polyelectrolytes is expected in the coming years to fulfill the ever-increasing demands for functional advanced materials.
